# Anti‐IgLON5 Disease with Isolated Hemichorea: A Case Report and Review of the Literature

**DOI:** 10.1002/mdc3.13614

**Published:** 2022-11-24

**Authors:** Anna Grossauer, Anna Hussl, Philipp Mahlknecht, Marina Peball, Anna Heidbreder, Florian Deisenhammer, Atbin Djamshidian, Klaus Seppi, Beatrice Heim

**Affiliations:** ^1^ Department of Neurology Medical University of Innsbruck Innsbruck Austria

**Keywords:** IgLON5, hemichorea, chorea, movement disorder, autoimmune encephalitis

## Abstract

**Background:**

Anti‐IgLON5 disease is an autoimmune encephalopathy with sleep disturbances as a hallmark in the majority of reported cases. Additional clinical symptoms are heterogenous and include movement disorders, bulbar dysfunction, autonomic disorders, and neurocognitive impairment.

**Case:**

Here, we report the case of an 87‐year‐old woman presenting with isolated progressive hemichorea. An extensive diagnostic work‐up revealed antibodies against IgLON5 in the serum. Neither history nor polysomnography (PSG) unveiled signs and features of sleep dysfunction typically reported in anti‐IgLON5 disease.

**Literature Review:**

In an extensive literature review we identified twelve other studies reporting about patients with confirmed anti‐IgLON5 disease and chorea as extrapyramidal movement disorder in their clinical phenotype. Subsequently, clinical characteristics of these patients were carefully evaluated.

**Conclusions:**

Our results support the diversity of clinical phenotypes in anti‐IgLON5 disease, adding isolated hemichorea to the spectrum of presenting symptoms. As sleep‐related disorders are often not the leading reason for consultation and only revealed by PSG examination, we suggest that screening for antibodies against IgLON5 should be considered in patients presenting with unexplained movement disorders, including isolated hemichorea.

IgLON5‐associated autoimmunity was first described as a central nervous system disorder with typical sleep disturbances.^1^ Reports of sleep disorders in anti‐IgLON5 disease particularly comprise sleep‐related vocalizations, movements or behaviors, and sleep disordered breathing with obstructive sleep apnea syndrome, stridor or central hypoventilation.^2^ Polysomnographic features show abnormal sleep initiation with undifferentiated non‐rapid eye movement (NREM) sleep or poorly structured N2 NREM sleep with parasomnias as well as rapid eye movement (REM) sleep behavior disorders.^3^ A recent review has shown that apart from sleep disorders the most frequent neurological symptoms in anti‐IgLON5 disease include bulbar dysfunction, gait abnormalities, movement disorders, autonomic dysfunction, and neurocognitive alterations in different combinations and severity, leading to distinct clinical phenotypes.^4^ Movement disorders observed in anti‐IgLON5 disease comprise chorea, tremor, dystonia, parkinsonism, and progressive supranuclear palsy‐like phenotypes.^5^ There is assumption that anti‐IgLON5 disease is a tau‐based disorder,^6^ albeit, recently a neuropathological case did not show phosphorylated tau brainstem deposits, suggesting that the tauopathy may be a secondary event in the disease.^7^ This observation is supported by in vitro findings advocating a link between IgLON5 autoantibodies and secondary neurodegeneration.^8^ Moreover, a significant association between the human leukocyte antigen (HLA) haplotype HLA‐DRB1*10:01‐DQB1*05:01 and anti‐IgLON5 disease was demonstrated lately, with patients being DRB1*10:01 positive presenting more often with sleep‐related symptoms and bulbar dysfunction.^9^

Here, we describe a case of anti‐IgLON5 disease manifesting with isolated progressive hemichorea lacking sleep‐related symptoms typically observed in anti‐IgLON5 disease in PSG. Furthermore, an extensive literature search was conducted to compare the clinical phenotypes of reported cases of anti‐IgLON5 disease presenting with chorea as extrapyramidal movement disorder.

## Case Series

An 87‐year‐old right‐handed woman of Caucasian descent presented in our outpatient department with slowly progressive hemichorea of the right side of the body that had started 2 years earlier (Video 1). Family, social, and medical history were unremarkable. No sleep problems, autonomic dysfunction or cognitive impairment were reported. In the neurological examination the patient was alert and well orientated, showed no signs of dysarthria and had an unremarkable oculomotor examination. Muscle tone and trophy were unremarkable, there was no bradykinesia or rigidity, but there were intermittent involuntary hyperkinetic movements of the right side of the body. Examination of gait revealed a mild instability due to the hemichorea also affecting the lower right limb.

**Video 1:** Overview of the patient's neurological examination demonstrating choreatic movements of the right side of the body.

Neuropsychological testing revealed normal cognitive functioning, and continuous blood pressure measurement in supine and standing position did not reveal any orthostatic dysregulation.

Cerebral magnetic resonance imaging (cMRI) did not show any structural abnormalities of the basal ganglia. Molecular‐genetic testing for Huntington's disease was negative.

Extensive peripheral blood tests including electrolytes, glucose, homocysteine, holotranscobalamine, vitamins B1, B3, and B12, folic acid, HbA1c, neuroakanthocythes, antinuclear, anticardiolipin, and antiphospholipid antibodies, antimyeloperoxidase, antithyroid peroxidase, antithyroglobulin antibodies and serum onconeural antibodies panel (anti‐Yo, anti‐Hu, anti‐Ri, anti‐CV2, anti‐Ma2, anti‐amphiphysin) were unremarkable. However, testing of antibodies directed toward neuronal surface antigens revealed positive anti‐IgLON5 serum antibodies. The patient declined lumbar puncture.

Polysomnographic examination was performed and revealed normal sleep architecture without the typical pattern of IgLON5‐associated sleep abnormalities. Repeated electroencephalographic examinations displayed no abnormalities. A whole body FDG‐PET‐CT scan did not show malignant lesions or inflammatory processes.

HLA‐class II typing was negative for the HLA‐DRB1*10:01 allele but revealed the HLA‐DQB1*05:03 allele.

Symptomatic treatment with tiapride was started showing a good therapeutic effect but was stopped by the patient due to the undesirable side effect of hair loss, resulting again in a worsening of chorea. Immunotherapy with corticosteroids as well as immunomodulating treatment with intravenous immunoglobulins was declined by the patient. A second therapeutic attempt with tiapride was started after some weeks, showing again a good therapeutic effect and was well tolerated by the patient this time. Hemichorea improved subjectively by 50% (as measured by a visual analogue scale) as it did 21 months after diagnosis, when the patient presented to the traumatology department due to a fracture of her right ankle. The patient declined further follow‐up visits because of her advanced age.

## Literature Review

We identified 12 case reports and case series^1^, ^10^, ^11^, ^12^, ^13^, ^14^, ^15^, ^16^, ^17^, ^18^, ^19^, ^20^ describing in summary 23 patients with anti‐IgLON5 disease presenting with chorea as extrapyramidal motor symptom in their clinical phenotype (Table 1). A sleep disorder was reported in all of these patients by history, however, confirmation of sleep abnormalities by PSG was only obtained in 14 of 23 patients (61%). In one study there was no detailed information on the detection of sleep disorders.^18^ Other symptoms appeared in heterogenous combinations and the vast majority of anti‐IgLON5 disease patients with chorea described in the literature suffered from a broad spectrum of complaints. Besides sleep disorders, the patients identified in our literature review particularly presented with gait impairment (16/23; 70%), bulbar symptoms (19/23; 83%), and neurocognitive or behavioral disorders (16/23; 70%). None of the cases identified in our literature review showed isolated hemichorea in their clinical phenotype.

**TABLE 1:** Cases of anti‐IgLON5 autoimmune encephalopathy with chorea as extrapyramidal movement disorder

Study	Age and Gender	Chorea	Sleep Disorder	Gait Impairment	Additional Movement Disorders	Oculomotor Symptoms	Bulbar Symptoms	Neurocognitive/behavioral Disorders	Autonomic Disorders
Cao et al., 2022	77, M	Lower face and neck	+*	+	Myoclonus of axial and upper limbs	+	+	+	−
Bhatti, 2022	75, F	Face and upper extremities	+	+	Oromandibular dystonia	+	−	−	−
Wang et al., 2021	62, M	Upper limbs	+	−	−	−	−	+	+
Aslam et al., 2020	58, M	Extremities, face and neck	+	−	−	−	+	+	+
Bhatia et al., 2020	58, M	Hands and toes	+*	+	Limb ataxia	−	+	+	−
Logmin et al., 2019	56, M	Hands	+*	−	Orofacial dyskinesias	+	+	+	−
Honorat et al., 2017	62, F	Choreoathetosis	+	+	Cervical dystonia	+	+	−	+
Honorat et al., 2017	59, F	NOS	+	+	−	−	+	+	+
Haitao et al., 2016	64, F	Jaw	+*	+	Parkinsonism	−	−	+	−
Simabukuro et al., 2015	71, F	Head and face	+*	+	Mild motor impersistance and hypotonia	+	−	+	−
Gaig et al., 2017**	NA	7/22 patients	7/7*	6/7	−	5/7	7/7	4/7	4/7
Sabater et al., 2014**	NA	4/8 patients	4/4*	3/4	Limb ataxia in 2/4 pat.	3/4	4/4	2/4	4/4
Strippel, Heidbreder, Mecklenbeck et al., 2022**	NA	2/11 patients	2/2	0/2	0/2	2/2	2/2	2/2	0/2

* Sleep disorders were confirmed by PSG examination, in the case series of Gaig et al. 5/7 patients received polysomnography.

** Case series.

Abbreviation: NOS, not otherwise specified.

## Discussion

Here, we report a case of anti‐IgLON5 disease presenting with progressive hemichorea, lacking the characteristic sleep pattern often associated with antibodies against IgLON5. To the best of our knowledge this is the first report of anti‐IgLON5 disease in a patient presenting with isolated hemichorea, and in which neither history nor PSG examination yielded sleep abnormalities that are usually associated with this disease. Typically, patients with anti‐IgLON5 disease and chorea suffer from additional clinical symptoms (Table 1). Therefore, our case further expands the spectrum of clinical phenotypes observed in anti‐IgLON5 disease with isolated hemichorea as presenting feature.

Typical sleep disturbances during NREM and REM sleep, including stridor and obstructive sleep apnea, snoring, insomnia and limb movements during sleep, associated with antibodies targeting the extracellular domain of the IgLON5 immunoglobulin‐like cell adhesion molecule, were described as the first hallmark of anti‐IgLON5 disease.^1^, ^2^ In the largest case series of patients with anti‐IgLON5 disease published to date, sleep disorders were present in nearly 90% of patients at the time of diagnosis.^5^ Sleep disorders, indeed, are a milestone of anti‐IgLON5 disease; however, this is not the main reason for an initial consultation in most patients; instead, gait problems, bulbar dysfunction or movement disorders are.^4^ Besides sleep disorders, gait problems, bulbar dysfunction and movement disorders, with chorea being reported in about a third of patients, dysautonomia, neurocognitive deficits and behavioral symptoms represent other common features in anti‐IgLON5 disease.^4^, ^5^

In a clinical follow‐up 21 months after diagnosis, our patient still had isolated hemichorea. The onset of sleep‐related disorders only months or years after initial symptoms is also a possible clinical course in anti‐IgLON5 disease.^21^, ^22^, ^23^, ^24^ However, as our patient did not show up for further follow‐up visits, we cannot determine whether sleep‐related symptoms may have occurred later in the disease course. Concerning the reports of sleep disorders, it is of utmost importance to note that sleep abnormalities are often not evaluated by the treating physician at initial presentation, which could result in an insufficient recognition of this characteristic feature in anti‐IgLON5 disease. Moreover, self‐reported perception of sleep often differs from objective sleep study measures^25^, ^26^ and there are reports of patients with anti‐IgLON5 disease, in which sleep abnormalities were only revealed by PSG.^27^, ^28^ Furthermore, PSG‐verified sleep disorders in anti‐IgLON5 disease can be more discrete than the typically described parasomnias or sleep‐related breathing disorders and only present as periodic limb movements during sleep.^29^

Concerning diagnostic features in anti‐IgLON5 disease, there is indeed a significant association between anti‐IgLON5 disease and the HLA‐DRB1*10:01‐DQB1*05:01 haplotype.^9^ Although our patient did not show the HLA‐DRB1*10:01 allele, she was positive for the HLA‐DQB1*05:03 allele, which has also been described in patients with anti‐IgLON5 disease.^9^ Intriguingly, patients being HLA‐DRB1*10:01 positive developed more frequently sleep or bulbar symptoms in one study.^9^ This finding might support the clinical phenotype observed in our patient not carrying the HLA‐DRB1*10:01 allele. Moreover, as the patient declined lumbar puncture testing of antibodies against IgLON5 was only performed in the serum and not in the CSF. However, this should not be regarded as a limitation to our case report as there is one study demonstrating that IgLON5 antibodies are more commonly found in the serum than in the CSF.^17^

Reports of anti‐IgLON5 disease questioned the relevance of immunotherapy, and most patients of the two earliest case series showed disease progression leading to death irrespective of immunotherapy.^1^, ^17^ However, to date there are reports of patients responding well to early immunomodulating strategies, including intravenous immunoglobulins, plasmapheresis, corticosteroids, rituximab or immunosuppressive agents like azathioprine or cyclophosphamide.^13^, ^15^, ^30^, ^31^ A systematic literature review including 46 patients with anti‐IgLON5 disease identified a response to immunotherapy in 43.4% of included cases,^32^ which is supported by the findings of a recently published study stating a response rate to first‐line immunotherapy in 41% of patients with relapse‐like acute to subacute exacerbation episodes.^31^ The same study^31^ also reported a stop in disease progression in 75% of patients treated with long‐term immunotherapeutic strategies, in which initiation during the first year after onset and low pre‐treatment neurofilament light chain were significant predictors for a better outcome. As a patient‐related limitation to our case report, therapeutic response to immunotherapy cannot be evaluated, as the patient declined immunotherapeutic strategies. Symptomatic treatment with tiapride, however, improved hemichorea considerably in our patient.

## Conclusion

This case report suggests that anti‐IgLON5 disease should be considered in patients presenting with adult onset hemichorea, even in the absence of typical sleep disorders associated with anti‐IgLON5 disease. In clinical routine, isolated hemichorea is usually associated with structural abnormalities in the basal ganglia. Therefore, an unremarkable cMRI in a patient with isolated hemichorea should be regarded as a red flag for a probable autoimmune etiology, including anti‐IgLON5 disease. As there is already some evidence for response to early immunotherapy,^13^, ^15^, ^30^, ^31^ we suggest that the indication for anti‐IgLON5 antibody screening should be extended and considered in patients even when sleep‐related symptoms are not present, especially when they present with bulbar symptoms, movement disorders, or neurocognitive impairment.

## Author Roles

(1) Research project: A. Conception, B. Organization, C. Execution.

(2) Manuscript preparation: A. Writing of the first draft, B. Review and Critique.

A.G.: 1A, 1C, 2A.

A.H.: 2B.

P.M.: 2B.

M.P.: 2B.

A.HE.: 2B.

F.D.: 2B.

A.D.: 2B.

K.S.: 1A, 1B, 1C, 2B.

B.H.: 1A, 1B, 1C, 2A, 2B.

## Disclosures

**Ethical Compliance Statement:** We confirm that we have read the Journal's position on issues involved in ethical publication and affirm that this work is consistent with those guidelines. Ethics approval of this manuscript was obtained by the ethics committee of the Medical University of Innsbruck (No. 1010/2022). Patient informed consent for video recording and publication was obtained as well.

**Funding Sources and Conflicts of Interest:** No specific funding was received for this work. The authors declare that there are no conflicts of interest relevant to this work.

**Financial Disclosures for the Previous 12 Months:** Anna Hussl, Philipp Mahlknecht, and Marina Peball declare that there are no additional disclosures to report. Anna Grossauer received travel grants from the Austrian Parkinson Society. Anna Heidbreder received honoraria for lectures or advisory board from Jazz Pharmaceuticals, UCB, Desitin, and travel support from Jazz Pharmaceuticals. Florian Deisenhammer has participated in meetings sponsored by or received honoraria for acting as an advisor/speaker for Almirall, Alexion, Biogen, Celgene, Genzyme‐Sanofi, Janssen, Merck, Novartis Pharma, and Roche. His institution has received research grants from Biogen and Genzyme Sanofi. He is section editor of the MSARD Journal (Multiple Sclerosis and Related Disorders). Atbin Djamshidian received honoraria from Novo Nordisk, Roche, Biogen und Abbvie. Klaus Seppi reports personal fees from Teva, UCB, Lundbeck, AOP Orphan Pharmaceuticals AG, Roche, Gruenenthal, Stada, Lucher Pharma, Biogen, BIAL, and AbbVie, honoraria from the International Parkinson and Movement Disorders Society, and research grants from FWF Austrian Science fund, Michael J. Fox Foundation and AOP Orphan Pharmaceuticals AG. Beatrice Heim reports honoraria from AOP orphan Pharmaceuticals AG.
